# A Neural Basis for the Acquired Capability for Suicide

**DOI:** 10.3389/fpsyt.2016.00125

**Published:** 2016-08-02

**Authors:** Gopikrishna Deshpande, Madhura Baxi, Tracy Witte, Jennifer L. Robinson

**Affiliations:** ^1^Department of Electrical and Computer Engineering, AU MRI Research Center, Auburn University, Auburn, AL, USA; ^2^Department of Psychology, Auburn University, Auburn, AL, USA; ^3^Alabama Advanced Imaging Consortium, Auburn University and University of Alabama Birmingham, Birmingham, AL, USA; ^4^Psychiatry Neuroimaging Laboratory, Department of Psychiatry, Brigham and Women’s Hospital, Harvard Medical School, Boston, MA, USA

**Keywords:** suicide, depression, gender difference, functional magnetic resonance imaging, diffusion tensor imaging, meta-analysis, meta-analytic functional connectivity, structural connectivity

## Abstract

The high rate of fatal suicidal behavior (SB) in men is an urgent issue as highlighted in the public eye *via* news sources and media outlets. In this study, we have attempted to address this issue and understand the neural substrates underlying the gender differences in the rate of fatal SB. The Interpersonal–Psychological Theory of Suicide has proposed an explanation for the seemingly paradoxical relationship between gender and SB, i.e., greater non-fatal suicide attempts by women but higher number of deaths by suicide in men. This theory states that possessing suicidal desire (due to conditions such as depression) alone is not sufficient for a lethal suicide attempt. It is imperative for an individual to have the acquired capability for suicide (ACS) along with suicidal desire in order to die by suicide. Therefore, higher levels of ACS in men may explain why men are more likely to die by suicide than women, despite being less likely to experience suicidal ideation or depression. In this study, we used activation likelihood estimation meta-analysis to investigate a potential ACS network that involves neural substrates underlying emotional stoicism, sensation-seeking, pain tolerance, and fearlessness of death, along with a potential depression network that involves neural substrates that underlie clinical depression. Brain regions commonly found in ACS and depression networks for males and females were further used as seeds to obtain regions functionally and structurally connected to them. We found that the male-specific networks were more widespread and diverse than the female-specific ones. Also, while the former involved motor regions, such as the premotor cortex and cerebellum, the latter was dominated by limbic regions. This may support the fact that suicidal desire generally leads to fatal/decisive action in males, while, in females, it manifests as depression, ideation, and generally non-fatal actions. The proposed model is a first attempt to characterize the neural networks underlying gender differences in SB. Future studies should examine the proposed network to better characterize and refine this network using tasks specifically targeted toward constructs underlying ACS.

## Introduction

Each year in the United States, over 40,000 individuals die by suicide (1). These figures do not include non-fatal suicide attempts, which are estimated to occur 25 times more frequently than fatal suicide attempts (1). One of the most well-established and, yet, paradoxical findings with regard to the epidemiology of suicidal behavior (SB) is that men are far more likely to die by suicide than women are (2), despite the fact that they are significantly less likely to experience depression [e.g., Ref. (3, 4)], suicidal ideation (5), and non-fatal suicide attempts (5). Although men tend to choose more lethal methods than women do (6, 7), a recent study has demonstrated that, even among those who choose the same method, men are more likely than women are to have a fatal outcome (8). Thus, method selection alone cannot explain the observed gender differences in fatal SB.

### Neural Mechanisms of Suicide

The neural basis of SB has been explored using structural (9–11), functional (12, 13), and metabolic imaging (14, 15). However, research on this topic often does not provide a satisfactory explanation for the observed sex differences in both non-fatal and fatal SB.

One conclusion from the existing literature is that prefrontal hypoactivity (16), which is modulated by decreased serotonin binding in the prefrontal cortex (17), is implicated in SB. However, hypoactivity in the prefrontal cortex is associated with a range of psychopathology, including depression (18), post-traumatic stress disorder (19), and schizophrenia (20). Thus, the specificity of this risk factor for SB is unclear. Additionally, there is evidence that women have decreased serotonin binding in the prefrontal cortex compared to men [e.g., Ref. (21, 22)]. This pattern suggests that prefrontal hypoactivity may explain the elevated risk for non-lethal SB in women. However, this risk factor does not provide a satisfactory explanation for the elevated risk for lethal SB in men. It is not surprising that the identified neural substrates for SB are plausible explanations for female vulnerability, as, virtually, all of the existing research has focused on non-fatal SB. In a recent review of functional and structural brain studies of SB (17), 21 of the 22 articles included were comparisons between non-fatal suicide attempters and controls. Given the association between female gender and non-fatal suicide attempts, conclusions from these research studies are not particularly informative regarding neural substrates that may explain the association between male gender and fatal suicide attempts.

Research on the neural substrates of fatal SB is hampered by the difficulty of conducting research on individuals who die by suicide. Indeed, brain imaging research on suicide decedents is impossible unless premorbid imaging data are available, as was the case in the sole imaging study that has examined suicide decedents (23). To date, this difficulty has been addressed using non-fatal suicide attempts as a proxy for fatal SB. This is problematic, as this approach will not uncover neural activations that distinguish fatal versus non-fatal SB, which may explain the gender paradox in SB that was presented above.

As an alternative to using non-fatal suicide attempts as a proxy for fatal suicide attempts, some have proposed the investigation of endophenotypes for SB [i.e., discrete, measurable traits that mediate the link between genetic risk and a particular form of pathology; Ref. (24)]. Given the heterogeneity of various forms of SB, the complex interplay between biological and environmental risk factors, and the difficulty in conducting research on fatal SB, focusing on endophenotypes may prove promising in elucidating biological risk factors for suicide. Several endophenotypes for suicide have been identified, to date [e.g., impulsive aggression, disadvantageous decision-making; Ref. (24)]. However, Courtet et al. (24) state the need for research that investigates gender differences in the neurobiology of SB, an aspect that has not been thoroughly investigated.

### The Interpersonal–Psychological Theory of Suicide

Although Courtet et al. (24) propose some promising endophenotypes for suicide, the existing literature on this topic is atheoretical. The benefits of grounding empirical research in theory are numerous; in this particular case, we propose that using a comprehensive account of suicide as a theoretical framework would integrate what is already known while fostering novel predictions. One theory that appears promising in this regard is the interpersonal–psychological theory of suicide (IPTS) (25, 26). According to the IPTS, even individuals who experience intense suicidal desire will not die by suicide without the fearlessness about death and pain tolerance necessary to endure the act of making a lethal suicide attempt. Together, fearlessness about death and physical pain tolerance comprise a novel construct first introduced by Joiner (25), known as the acquired capability for suicide (ACS). According to the IPTS, suicidal desire stems from the simultaneous presence of thwarted belongingness (i.e., loneliness and lack of reciprocal care) and perceived burdensomeness (i.e., feeling like a liability on others and self-hatred). It is only when a desire for suicide simultaneously occurs with the ACS that a lethal suicide attempt is even possible. The IPTS offers the following explanation for gender differences in non-fatal and fatal SB: women are more likely to experience thwarted belongingness and/or perceived burdensomeness and men are more likely to acquire the capability for suicide – a proposition that has borne out in the behavioral literature [e.g., Ref. (27–30)]. Consequently, men who experience suicidal desire may be more likely to have a fatal outcome than women with similar levels of suicidal desire.

There are two main explanations for why men may have higher ACS than women do. First, according to the IPTS, the capability for lethal self-harm is acquired primarily through exposure to life experiences that are painful and provocative, which result in habituation to fear of death and/or physical pain. Many such experiences (e.g., combat exposure, impulsive/aggressive behaviors) are more common among men. Second, the IPTS allows for the possibility that various neurobiological and temperamental factors may make an individual more likely to acquire the capability for suicide over the course of his/her lifetime (26). A recent study (30) examined sensation-seeking and emotional stoicism as potential temperamental characteristics that explain the relationship between gender and both facets of ACS. Across two large, independent samples, sensation-seeking fully accounted for the relationship between gender and fearlessness about death, and stoicism fully accounted for the relationship between gender and physical pain insensitivity. Thus, these temperamental characteristics may explain the observed gender differences in the ACS and, therefore, greater likelihood of death by suicide among men.

Witte et al.’s (30) findings, when viewed within the purview of IPTS, suggest a possible brain network that may explain the biological basis for the gender differences in lethal SB. We hypothesize that this network would involve neural substrates that underlie emotional stoicism, sensation-seeking, pain tolerance, and fearlessness about death, all of which may be considered endophenotypes for fatal suicide attempts. Our proposed brain network is based on a body of literature, suggesting that these constructs are interconnected. Numerous studies have demonstrated an association between male gender and both stoicism and sensation-seeking (31–35). Additionally, several studies have established the link between stoicism and pain tolerance [e.g., Ref. (30, 36–39)]. Likewise, sensation-seeking has been found to be an important correlate of ACS, as two studies have demonstrated associations between sensation-seeking and the ACS (30, 40). Given the overlapping theoretical constructs, the current study represents the first investigation of the neural substrates that may underlie gender differences in lethal SB.

As noted above, the existing research on the neural basis of SB is limited and is largely focused on factors that explain the increased risk for non-fatal SB seen in women, without providing an adequate explanation for the increased risk for death by suicide seen in men. By parsing out suicidal desire and capability for suicide, the IPTS offers a useful, theoretically driven framework, suggesting the possibility of separate neural substrates underlying the theoretical constructs. In this study, we hope to address the two aforementioned challenges in this area of research by distinguishing the neural substrates for ACS from neural substrates relevant to suicidal desire and attempting to explain the higher suicide mortality seen in men in terms of gender differences of underlying neural substrates. In order to do so, we conducted two separate activation likelihood estimation (ALE) meta-analyses. The first focused on our proposed ACS brain network by finding brain regions commonly activated by at least two of the four tenets of ACS: emotional stoicism, sensation-seeking, pain tolerance, and fearlessness of death. Our second meta-analysis focused on brain regions activated by clinical depression, since it is intricately linked to suicidal desire. The intersection of these two followed by meta-analytic connectivity modeling provided us with an ACS–depression network, which may underlie lethal suicide attempt and which is distinct in males and females. Further, we performed structural connectivity analysis using diffusion tensor imaging (DTI) for demonstrating that this ACS–depression network has different structural connectivity patterns in males and females. We show that the meta-analyses coupled with insights from DTI leads to testable hypotheses regarding the neural basis of IPTS and the ACS.

## Materials and Methods

### Activation Likelihood Estimation

Activation likelihood estimation algorithm (41–43) was used to investigate brain regions mediating gender differences underlying ACS and depression. ALE is a widely used probabilistic approach for coordinate-based meta-analysis. Importantly, ALE accounts for the spatial uncertainties associated with different subjects and brain templates. Here, we present a brief overview of the general procedure involved while performing meta-analysis based on ALE. In the following section, we explain our specific analyses. In the ALE approach, every focus that was reported to be activated in an experiment yields an estimated 3D probability distribution with the center of distribution being at the focus. The ALE scores for each voxel are calculated by the union of activation probabilities for each voxel. The activation probability of a given focus at a voxel is calculated using a Gaussian probability function *P*.

$$P=\frac{e^{\frac{-d^{2}}{2\sigma^{2}}}}{{\left(2\pi \right)}^{0.5}\;\sigma }$$

where *d* is the Euclidean distance from center of voxel to particular foci, σ is the SD of the probability distribution. The σ values are calculated using the Euclidean distance between the same focus in different subjects. After obtaining the ALE scores of all voxels present in the brain, they are then compared with a null distribution. The null distribution is obtained by calculating ALE scores of voxels when there is no biological activity in the brain. By comparing the activation-related ALE score of voxels with the null distribution, a thresholded activation map can be generated (41–43).

### Meta-analytic Connectivity Modeling: An ALE Meta-analysis

We searched the BrainMap database for papers coded with specific search criteria, described below, using the Sleuth search portal (44–46). The BrainMap database archives whole-brain coordinates from functional neuroimaging studies, using a rigorous coding scheme (46, 47). Coordinates of activation from contrasts meeting our criteria were then downloaded, and meta-analytic statistics were computed using GingerALE software (41–43) to determine regions of convergence among our search set using the ALE algorithm described in the previous section. Resultant ALE maps were thresholded with a minimum cluster size of 100 mm^3^ and a *p*-value of 0.05 and corrected for false positives using false discovery rate. Robinson et al. (48, 49) coined the term “meta-analytic connectivity modeling,” which is based on the assumption that voxels that are statistically coactivated by a given condition across many different experiments must be functionally connected (48, 49). Here, we used this concept to find functional networks underlying ACS and depression in males and females. All searches had the basic criteria of including only activations for both ACS and depression and only normal subjects in the case of ACS whereas only depressed in the case of Depression. All searches were performed for males and females separately. In order to form a functional neural network for ACS, different ALE meta-analyses were performed using search criteria related to (i) emotion (i.e., all aforementioned search criteria plus “Experiments – Behavioral domain – Emotion – All subtypes” giving us, for males: 446 experiments, 2145 subjects and for females: 355 experiments, 149 subjects, to be used for ALE meta-analyses); (ii) pain processing (i.e., all basic search criteria plus “Experiments – Behavioral domain – Perception – Somes thesis Pain,” giving us, for males: 79 experiments, 372 subjects and for females: 34 experiments and 178 subjects, to be used for ALE meta-analyses); (iii) sensation-seeking (i.e., all basic search criteria and experiments with paradigm class as reward, given the intimate association between sensation-seeking and the brain’s reward system, giving us, for males: 127 experiments and 621 subjects and for females: 35 experiments and 174 subjects, to be used for ALE meta-analyses); and (iv) fear (i.e., all basic search criteria in addition to “Experiments – Behavioral domain – Emotion – Fear,” giving us, for males: 54 experiments and 318 subjects and for females: 32 experiments and 231 subjects, to be used for ALE meta-analyses). Each of these searches was run separately. The above search criteria are motivated by the four main constructs underlying IPTS as outlined in the Section “Introduction,” i.e., emotional stoicism, sensation-seeking, pain tolerance, and fearlessness of death. It is noteworthy that using these exact terms in the Sleuth search would give very few or no relevant papers. Therefore, we searched for broader conceptualizations of these constructs. For example, we believe that the extent of activation in the fear-related regions might be an important factor modulating fearlessness of death, and hence, we used “Experiments – Behavioral domain – Emotion – Fear” as the search criterion. The ACS was proposed with two constructs, pain tolerance and fearlessness of death. However, a recent study (30) examined sensation-seeking and emotional stoicism as potential temperamental characteristics that explain the relationship between gender and both facets of ACS. Therefore, as the prevalence of at least two of the four constructs underlying IPTS might account for ACS, we deemed all voxels that were activated by two or more of the search conditions to represent the functional neural network in males and females separately for ACS (we will call this “ACS network”).

**Figure 1:**
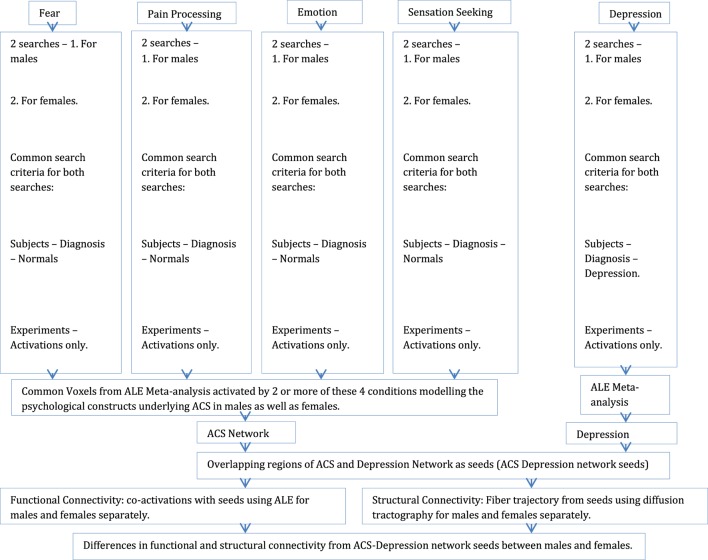
**A schematic illustration of the entire procedure involving ALE meta-analysis, meta-analytic functional connectivity modeling, and structural connectivity analysis**.

In order to obtain the functional neural network for depression, ALE meta-analyses were performed using search criteria related to depression. Two Sleuth searches were performed, one for males and the second for females. Common search criteria used for both of the searches were subjects diagnosed with depression  (Subjects – Diagnosis – Depression) and experiments showing only activations as results (Experiments – Activations only), giving us, for males: 31 experiments and 112 subjects and for females: 29 experiments and 149 subjects, to be used for ALE meta-analyses. Additional criterion used for the first search was only male subjects (Subjects – Gender – Males only) and the second search was for only female subjects (Subjects – Gender – Females only). The resultant ALE map from this search was interpreted as a representation of the functional neural network in males and females, separately underlying depression (we will call this “depression network”). Figure 1 describes the steps involved in forming the ACS and depression networks.

Acquired capability for suicide and depression networks were examined to identify overlapping voxels, which may be indicative of common neural substrates underlying ACS and depression in males and females. These common voxels (we will call these “ACS–depression network seeds”) were then used as ROIs in a subsequent ALE-based meta-analysis, wherein these seed locations were used for obtaining voxels coactivated by them, and hence, by inference, functionally connected to them. Further, we investigated whether the functional network obtained by ACS–depression network seeds using meta-analytic connectivity modeling (we call this “ACS–depression network”) were different in males and females.

### Structural Connectivity Using Diffusion Tensor Imaging

To investigate the structural basis of the functional networks derived through meta-analysis, DTI techniques were used. White matter axonal tracts, from the ACS–depression network seed voxels identified previously, were calculated using diffusion-weighted data in order to determine the regions structurally connected to them. Diffusion-weighted data were acquired from 31 healthy individuals using Siemens 7T MAGNETOM scanner (26 right-handed, 12 males, 19 females, M ± SD = 21.13 ± 1.43). The study was approved by the Institutional review board at Auburn University and all human subjects provided informed consent. A high resolution DTI scan (40 slices, 2 mm^3^ isotropic voxels, TR/TE: 5200/94 ms, base/phase resolution 122/100%, GRAPPA acceleration factor of 3, *b* = 0 and 1000, 30 directions, 3 averages, collected in an interleaved fashion) was acquired. All diffusion-weighted images were skull-stripped using tools provided in FSL software (50) and manually checked to ensure accuracy. Probabilistic diffusion tractography was carried out as described previously (51–53), using a probability density function that was created at each voxel on the principal fiber direction. Connectivity probabilities were estimated between the seed voxels and target voxels (i.e., the rest of the brain) by repeatedly sampling connected pathways through the probability distribution function. The differences in the white matter pathways originating from ACS–depression network seeds in males and females were examined. Figure 1 illustrates the entire analysis procedure.

## Results

### The ACS and Depression Networks

**Figure 2:**
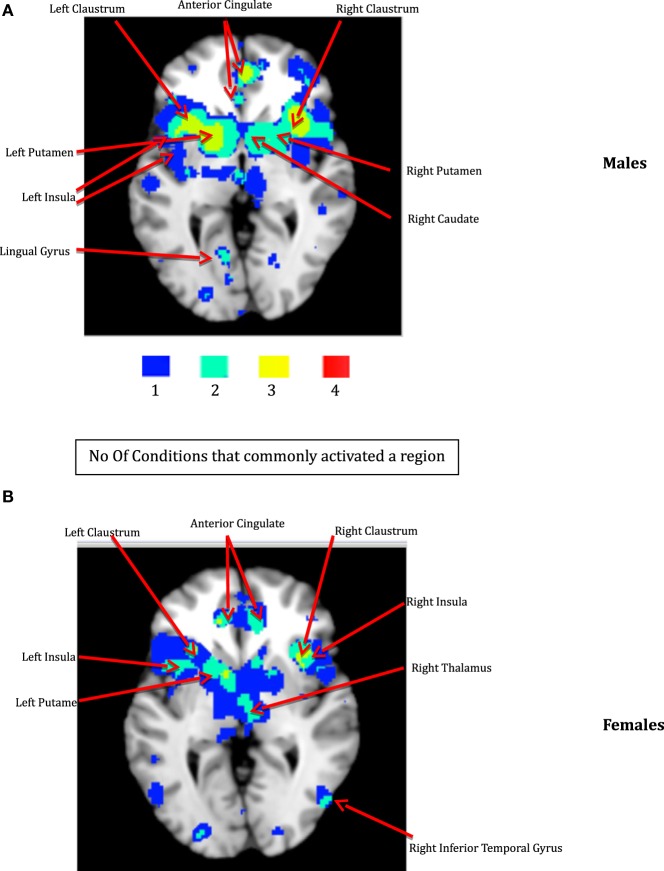
**The ACS network for males (A) and females (B)**. The color bar illustrates the color scheme used for depicting the regions that were commonly activated by just one, two, three, or four of the following conditions – emotion, pain processing, sensation-seeking, and fear. Voxels activated by any one of the above mentioned four conditions are shown in blue, by any two of the above conditions are shown in aquamarine, and by any three of the four conditions in yellow, respectively. Regions represented by aquamarine and yellow colors together form the ACS network.

**Figure 3:**
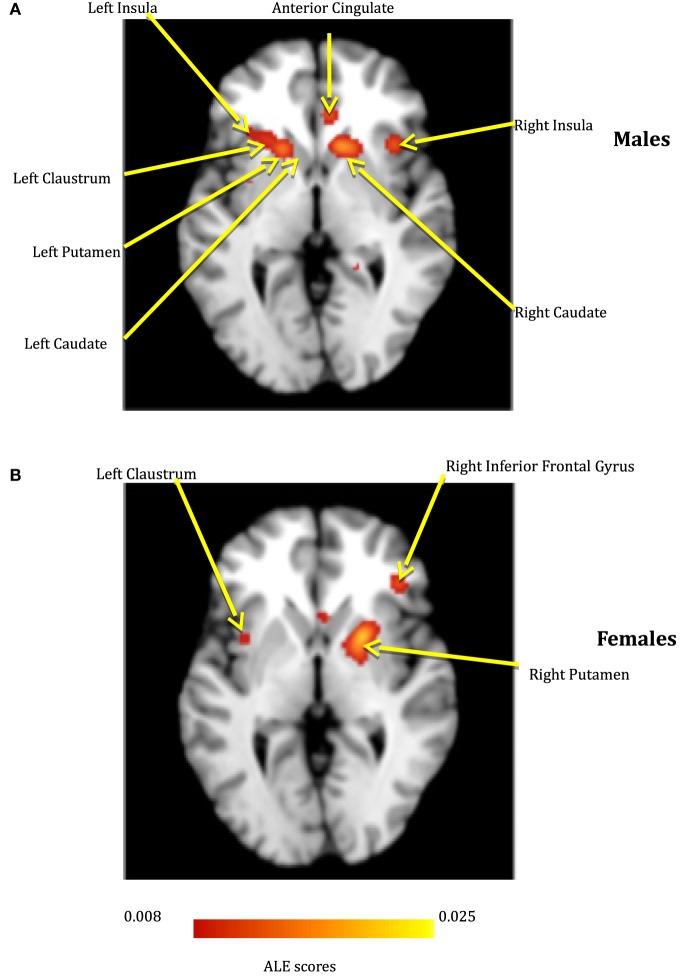
**The depression network for males (A) and females (B)**.

The thresholded ALE maps obtained from conducting ALE meta-analysis on the aforementioned four tenets of ACS, emotional stoicism, sensation-seeking, pain tolerance, and fearlessness of death, were overlaid on the same anatomical image for males and females separately. The regions that were commonly activated by at least two of the four conditions were hypothesized to represent the ACS network for males as well as females (Figure 2). Similarly, regions constituting the functional network underlying depression, obtained from ALE-based meta-analysis performed for depression are shown in Figure 3 for males and females. Table 1 provides list of the major regions in the ACS network in males and females, separately showing regions of overlap as well as gender-specific activations. The regions that were common to the functional networks underlying depression in males and along with those that were specific to males or females are listed in Table 2. Note that ALE values are provided in Table 2 and not in Table 1, as the latter is an intersection map obtained from four different primary ALE analyses.

**Table 1:** **Activation statistics in males and females corresponding to the ACS network**.

Lobe	Region	BA	Males			Females		
			*x*	*y*	*z*	*x*	*y*	*z*
**Convergent seeds**								
Sub-lobar	Right caudate	Caudate body	8	10	4	8	12	6
	Right putamen (lentiform nucleus)		21	4	4	17	6	6
	Right claustrum		29	16	4	33	20	3
	Right insula		41	14	4	36	19	3
	Left putamen (lentiform nucleus)		−18	6	2	−25	−3	−9
	Left claustrum		−29	17	2	−30	12	6
	Left insula	13	−40	13	2	−36	11	6
	Right thalamus		8	2	4	3	−14	2
**Male-specific network**								
Sub-lobar	Left caudate	Caudate body		−9		11		2
Frontal	Right precentral gyrus	44		48		12		4
	Left precentral gyrus	6		−42		0		35
	Right mid frontal gyrus	10, 9		35		41		13
				40		17		25
Posterior	Right cerebellum declive			33		−71		−17
	Left cerebellum declive			−33		−63		−18
Limbic	Right anterior cingulate	32		6		45		3
	Right cingulate gyrus	23		5		−28		26
Occipital	Right lingual gyrus	17		21		−87		8
	Left lingual gyrus			11		−63		2
**Female-specific network**								
Sub-lobar	Left lat glob pallidus (lentiform nucleus)			−19		−4		−9
	Amygdala			−20		−7		−13
	Right lat glob pallidus (lentiform nucleus)			18		1		−10
Limbic	Left cingulate gyrus	32, 24		−3		12		42
				−3		9		42
	Left anterior cingulate	32		−12		39		0
	Right anterior cingulate	24		7		37		4
Parietal	Left postcentral gyrus	40		−54		−27		20
Occipital	Right inf temporal gyrus	37		48		−69		2

*BA, Brodmann area*.

**Table 2:** **Activation statistics in males and females corresponding to the Depression network**.

Lobe	Region	BA	Males			Females			ALE	
			*x*	*y*	*z*	*x*	*y*	*z*		
**Convergent regions**										
Sub-lobar	Right putamen (lentiform nucleus)		28	−4	8	22	8	2	0.015	0.020
	Left putamen (lentiform nucleus)		−26	−2	8	−24	−4	10	0.014	0.024
						−22	−2	−6		0.012
						−26	10	6		0.009
	Left claustrum		−24	20	4	−28	8	−8	0.012	0.014
	Right insula	13	40	−18	16	44	−38	18	0.025	0.010
			38	18	4				0.015	
			38	4	10				0.008	
	Left insula	13	−50	−18	24	−36	6	4	0.012	0.010
	Right caudate	Caudate head	14	16	0	2	16	2	0.019	0.009
										
Limbic	Left cingulate gyrus	23	−6	−32	28	−8	−12	30	0.014	0.013
**Female-specific network**										
Frontal	Right inferior frontal gyrus	47		40		32		0		0.013
				18		20		−16		0.011
				18		30		−2		0.009
				24		30		−4		0.008
	Left mid frontal gyrus	9		−50		14		26		0.010
Limbic	Left anterior cingulate	32		−4		20		−8		0.012
	Left cingulate gyrus	23		−8		−12		30		0.013
	Right posterior cingulate	23		4		−28		22		0.024
	Right anterior cingulate	24, 32, 25		6		32		4		0.015
				2		26		−8		0.013
				2		2		−4		0.010
	Right cingulate gyrus	31, 24		12		−40		28		0.010
				12		8		26		0.014
	Right parahippocampal gyrus	35, 36		24		−20		−18		0.018
				28		−28		−10		0.014
	Left parahippocampal gyrus	28, 19		−20		−18		−14		0.013
				−24		−42		−2		0.010
Temporal	Left middle temporal gyrus	39		−44		−62		22		0.010
Anterior	Right cerebellum anterior lobe: dentate			12		−52		−22		0.009
Sub-lobar	Left lat glob pallidus (lentiform nucleus)			−20		−3		6		
**Male-specific network**										
Frontal	Left precentral gyrus	9, 6		−36		8		38		0.023
				−37		5		35		
	Right precentral gyrus	6		38		2		34		0.011
										
	Right mid frontal gyrus	46, 8, 9		42		28		18		0.014
				28		12		38		0.013
				39		18		23		
	Left inferior frontal gyrus	45		−32		24		4		0.010
Parietal	Left supramarginal gyrus	40		−38		−44		34		0.020
	Left inferior parietal lobule	40		−46		−42		28		0.017
	Right inferior parietal lobule	40		42		−34		34		0.015
Sub-lobar	Left caudate	Caudate head		−16		16		2		0.016
	Right caudate	Caudate body		16		−18		26		0.011
	Right claustrum			36		−4		−4		0.011
	Right thalamus			4		−4		6		0.011
Temporal	Left superior temporal gyrus	13		−36		−26		8		0.011
Occipital	Right lingual gyrus	30		18		−42		0		0.009
Limbic	Right cingulate gyrus	23		5		−28		28		

*BA, Brodmann area*.

### The Overlap between ACS and Depression Networks

**Figure 4:**
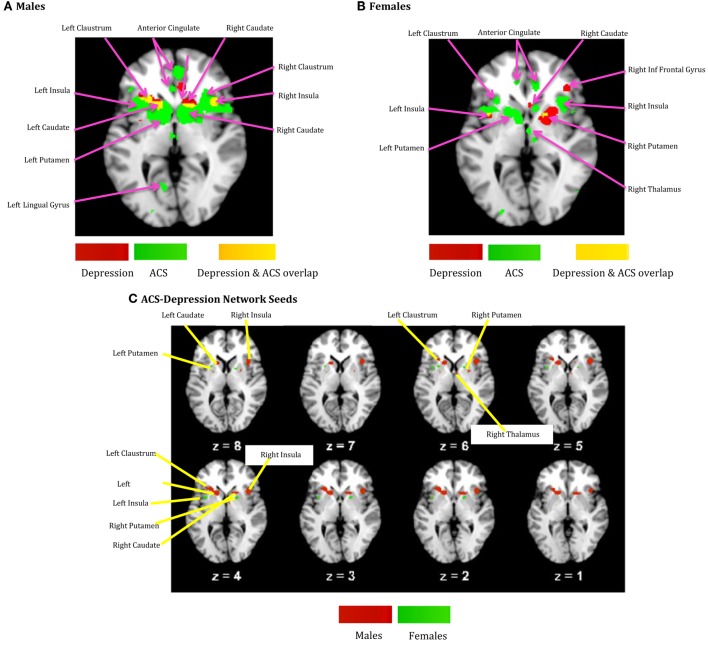
**(A)** ACS and depression networks identified in males, both overlaid on same anatomical image. **(B)** ACS and depression networks identified in females, both overlaid on same anatomical image. **(C)** Voxels commonly found in the ACS and depression networks identified in males and females, overlaid on a single anatomical image. These form the ACS–depression network seeds.

The voxels identified in the ACS and depression networks in males and females from Figures 2 and 3, respectively, were overlaid on a single anatomical image as shown in Figure 4, to investigate common neural substrates underlying ACS and depression. The overlap between ACS and depression networks in males consisted of left precentral gyrus, bilateral putamen, left claustrum, bilateral caudate, right cingulate gyrus, right Insula, right middle frontal gyrus (also referred to as dorsolateral prefrontal cortex), and right thalamus. Likewise, the overlap between ACS and depression networks in females consisted of bilateral putamen, left lateral globus pallidus, and left insula. These activations in the males and females form the ACS–depression network seeds, which were subsequently used in order to determine meta-analytic functional connectivity and DTI-based structural connectivity. As noted before, since the depression network was assumed to represent a network that may underlie suicidal desire, the overlap between ACS and Depression networks may form a basis for lethal SB. Putamen was commonly activated in ACS–depression Network in males as well as females. The ACS–depression network seeds organized by lobes and weighted centers of seeds that were obtained in males and females are enlisted in Table 3.

**Table 3:** **Seeds corresponding to ACS–depression network (Figure 4C)**.

Lobe	Region	BA	Males			Females		
			*x*	*y*	*z*	*x*	*y*	*z*
**Convergent seeds**								
Sub-lobar	Left putamen (lentiform nucleus)		−19	14	4	−26	9	7
	Right putamen (lentiform nucleus)		24	1	7	17	7	4
**Female-specific seeds**								
Sub-lobar	Left insula	13		−37		7		5
	Left lat glob pallidus (lentiform nucleus)			−21		0		6
**Male-specific seeds**								
Sub-lobar	Left claustrum			−27		21		4
	Left caudate	Caudate body		−16		15		4
	Right caudate	Caudate body		14		15		4
	Right insula	13		37		18		5
	Right thalamus			5		−2		6
Frontal	Left precentral gyrus	6		−40		2		35
	Right middle frontal gyrus	9		40		18		23
Limbic	Right cingulate gyrus	23		4		−28		28

### Functional and Structural Connectivity

**Figure 5:**
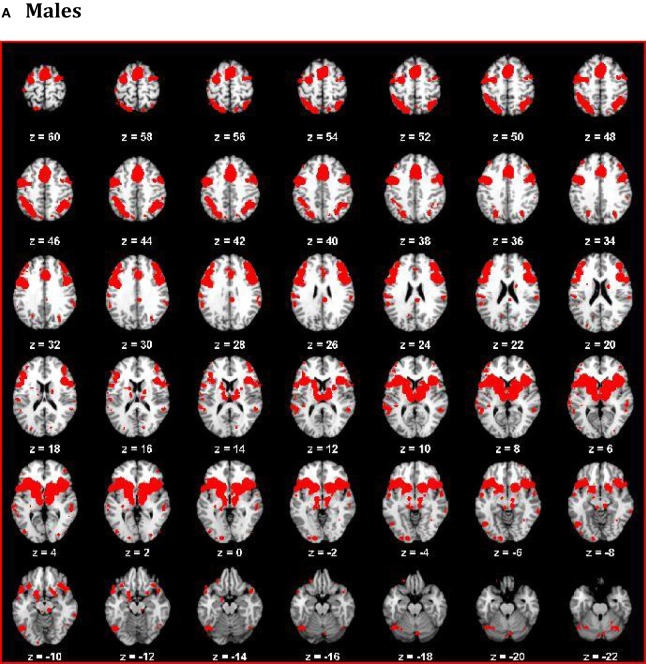

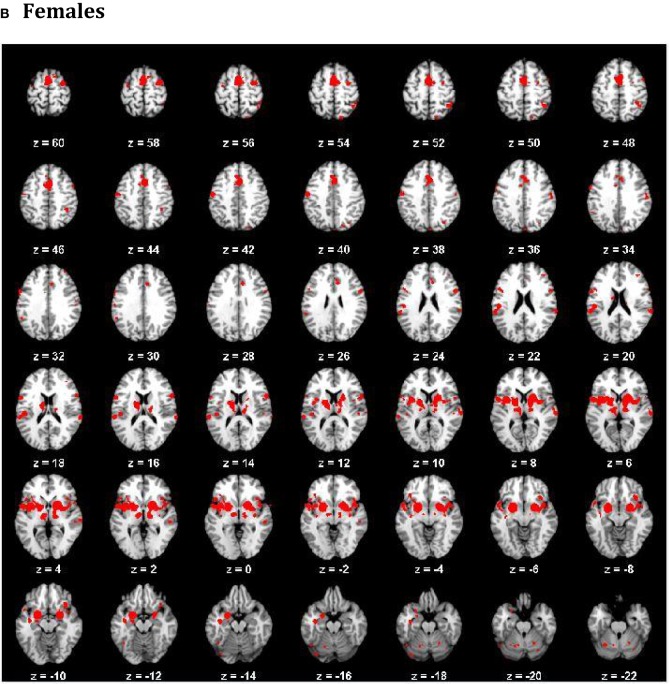
**ACS–depression network in males (A) and females (B) obtained by finding voxels coactivated by the ACS–depression network seeds found in Figure 4**.

**Figure 6:**
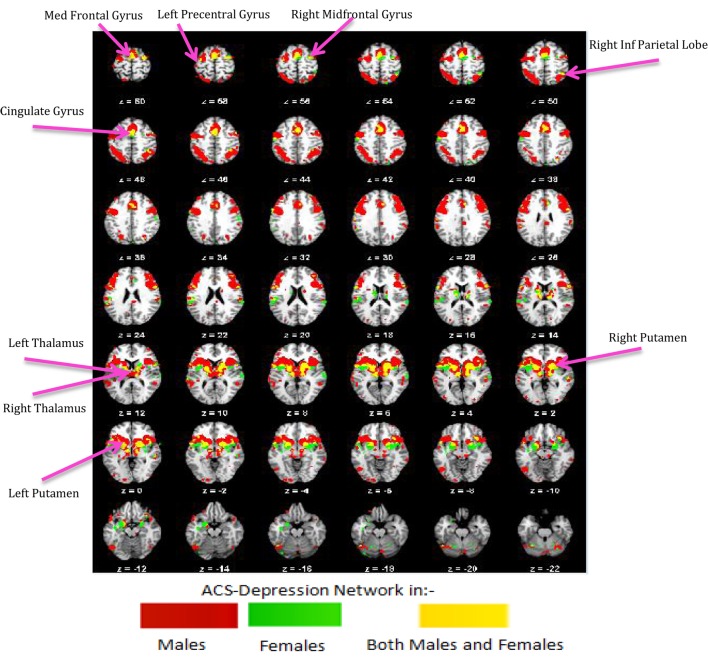
**ACS–depression network, which was obtained separately in males and females, is overlaid on a single anatomical image**. Green represents the ACS–depression network in females, red in males, and yellow represents their overlap. Only regions of overlap are labeled in the figure while Table 5 provides labels of regions, which were exclusively coactivated with ACS–depression network seeds only in males and females.

We obtained voxels coactivated by the ACS–depression network seeds (Figure 4) separately in males and females, using ALE-based meta-analytic connectivity modeling. The corresponding results, shown in Figures 5 and 6, indicate that, even though the ACS–depression network in males and females underlie a common neurophysiological framework, there are both commonalities and differences in their underlying neural substrates. Major brain regions, which were commonly coactivated by the ACS–depression network seeds in males and females, are listed in Table 4, while gender-specific coactivations are listed in Table 5. This provides an exploratory model for differentiating the neural basis of lethal suicide attempts between males and females.

**Table 4:** **Major regions that demonstrated meta-analytic functional connectivity to the ACS–depression seeds in both males and females**.

Lobe	Region	BA	*x*	*y*	*z*
Sub-lobar	Left putamen (lentiform nucleus)		−19	4	4
	Left lat glob pallidus (lentiform nucleus)		−19	−3	4
	Left insula	13	−38	10	4
	Left thalamus		−10	−16	4
	Right putamen (lentiform nucleus)		23	5	4
	Right lat glob pallidus (lentiform nucleus)		18	0	4
	Right insula	13	42	8	4
	Right caudate	Caudate body	13	8	4
	Right thalamus		15	−15	4
	Right claustrum		31	24	−7
Frontal	Left precentral gyrus	44	−51	8	4
	Left med frontal gyrus	6, 32	−3	3	58
			−2	13	43
	Right med frontal gyrus	6, 32	4	5	58
			6	15	43
	Right midfrontal gyrus	6	28	−3	60
	Right inf frontal gyrus	44	54	11	15
Limbic	Right cingulate gyrus	32, 24	2	16	41
			2	14	45
	Left cingulate gyrus	32, 24	−2	23	41
			−3	10	38
Parietal	Right inf parietal lob	40	39	−46	50
Anterior	Left cerebellum culmen		−29	−60	−26

**Table 5:** **Male- and female-specific major regions exclusively coactivated with ACS–depression network seeds**.

Lobe	Regions	BA
**Male specific-regions**		
Sub-lobar	Left claustrum	
	Left putamen (lentiform nucleus)	
	Left caudate	Caudate body
	Right thalamus	
	Right putamen (lentiform nucleus)	
	Right insula	13
	Right caudate	Caudate body
	Left thalamus	
	Left insula	13
	Right caudate	Caudate head
	Right claustrum	
Frontal	Left precentral gyrus	6
	Right mid frontal gyrus	9
	Left precentral gyrus	44
	Left mid frontal gyrus	6, 9, 46
	Left inf frontal gyrus	9, 45, 13
	Right inf frontal gyrus	9
	Right precentral gyrus	6, 9
	Right med frontal gyrus	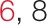
	Right sup frontal gyrus 6	
Limbic	Right cingulate gyrus	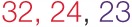
Parietal	Left inf parietal lob	40
	Left angular gyrus	39
	Left precuneus	7, 19, 39
	Left sup parietal lob	7
	Left supramarginal gyrus	40
	Right sup parietal lob	7
	Right precuneus	7
	Right inf parietal lob	40
Temporal	Left fusiform gyrus	37
	Left sup temporal gyrus	22
	Left mid temporal gyrus	21
	Right sup temporal gyrus	41, 22
	Right mid temporal gyrus	21
Anterior	Right cerebellum culmen	
Posterior	Right cerebellum pyramis	
Occipital	Left mid occipital gyrus	37
**Female-specific regions**		
Sub-Lobar	Left putamen (lentiform nucleus)	
	Left lat glob pallidus (lentiform nucleus)	
	Left claustrum	
	Right putamen (lentiform nucleus)	
	Right insula	13
	Right claustrum	
	Left insula	13
	Left thalamus	
	Left caudate	Caudate body
Frontal	Left precentral gyrus	44
	Right med frontal gyrus	6
	Right mid frontal gyrus	6
Temporal	Right sup temporal gyrus	42
	Right transverse tem gyrus	41

*Regions that contained some overlapping area between males and females but also were observed to contain some area specific to male/female network are mentioned in red, regions consisting of ACS–depression seeds are mentioned in violet and regions satisfying both the aforementioned criteria are mentioned in green*.

**Figure 7:**
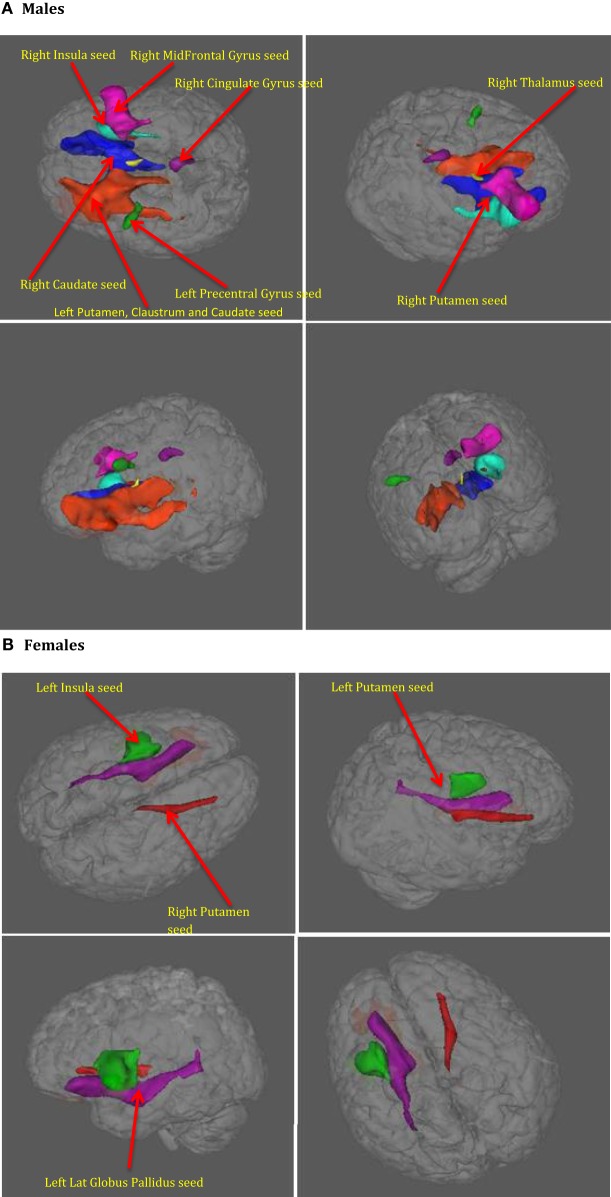
**The axonal trajectories derived from the ACS–depression network seeds defined by coactivated voxels from ACS and Depression networks in males (A) as well as females (B)**. The four panels in **(A,B)** are the different views of the same figure. **(A)** Yellow – fibers from right thalamus, green – fibers from left precentral gyrus, dark blue – fibers from right caudate, purple – fibers from right cingulate gyrus, light blue – fibers from right insula, pink – fibers from right mid frontal gyrus, red – fibers from right putamen, orange – fibers from left putamen, claustrum, caudate. **(B)** Red – fibers from right putamen, green – fibers from left insula, blue – fibers from left putamen (hidden inside the green fibers from left insula, so not seen in the figures), purple – fibers from left globus pallidus.

Further, we performed axonal fiber tractography using the ACS–depression network seeds to demonstrate the distinct structural connectivity likely involved in the ACS–depression network in males and females. The axonal trajectories derived from the ACS–depression network seeds in males and females are shown in Figure 7. Further, a 3D rendering of the trajectories is shown in Videos S1 and S2 (corresponding to males and females) in Supplementary Material accompanying this paper, in order to give a better understanding of the projections from the seeds.

**Figure 8:**
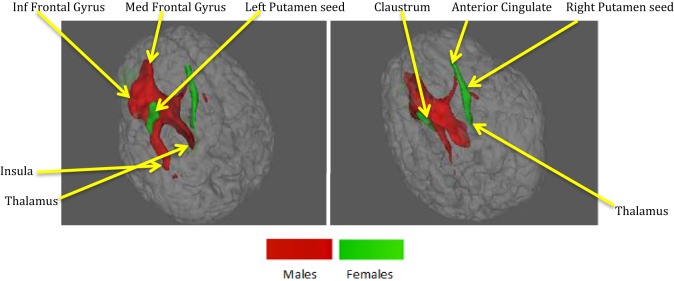
**Fibers from seeds in left and right putamen in males and females both overlaid on a single anatomical**. Red – fibers in males, green – fibers in females. Both panels represent different views of the same figure.

It can be observed from Figures 4 and 7 and Table 3 that ACS–depression network seeds in the left and right putamen are present in both males and females. Given the role of putamen in the hate circuit (54), it may mediate gender differences in lethal SB (more on this in the Section “Discussion”). In order to better understand and demonstrate the differences in structural connectivity between males and females involved in ACS–depression network, just the fiber trajectories from these seeds in both males and females were overlaid on the same anatomical image in Figure 8. The fiber trajectories from the left putamen seed in males, projected toward left insula, medial frontal gyrus, thalamus, and premotor cortex. Whereas in females, the fiber projections from left putamen did not travel farther while that from the right putamen seed projected to the anterior cingulate and thalamus. This qualitative depiction of structural connectivity demonstrates that the ACS–depression network in males might cover a vast expanse of cortical and sub-lobar brain regions, compared to females.

## Discussion

One of the most well-established findings with regard to SB is that men are far likelier to engage in fatal SB, whereas women are far likelier to engage in non-fatal SB. Most existing functional and structural studies of suicide are focused on non-fatal SB (17); as such, they do not necessarily provide information about neural substrates involved in fatal SB and, therefore, cannot explain the so-called gender paradox of suicide. The goal of this study was to address this limitation in the literature. Given the challenges inherent in studying the neural basis for fatal SB, we used the IPTS (25, 26) as a theoretical foundation for identifying potential endophenotypes of fatal SB. On the basis of prior research (30), we investigated several psychological constructs relevant to the ACS (i.e., emotional stoicism, sensation-seeking, pain tolerance, and fearlessness about death). Gender differences in neural networks that underlie these endophenotypes may eventually help to explain the gender paradox of SB. To accomplish our goal, we conducted an exploratory investigation of the neural mechanisms that are differentially activated by the psychological/psychiatric constructs in the ACS and depression in males and females. As noted before, since the depression network was assumed to represent a network that may underlie suicidal desire, the overlap between ACS and depression networks may form a basis for lethal SB. Here, we have demonstrated that meta-analysis and meta-analytic connectivity modeling can be used to develop neural models and testable hypotheses regarding the gender paradox of SB.

Our research has identified a preliminary network of regions commonly activated by two or more psychological constructs underlying the ACS in males and females separately. Given that most functional and effective connectivity models require the definition of *a priori* ROIs, identifying the neural nodes associated with ACS in a sensitive and robust fashion represents a key advancement for future experimental studies that can examine gender differences in the neural connectivity of these networks. Second, we found that the regions corresponding to both the ACS and depression networks have significantly different foci in males and females, which implies potential for distinct functional and structural connectivity differences. The proportional contribution of each of the regions in the ACS and depression networks to the individual psychological constructs of the IPTS may provide a gender-specific, multidimensional imaging biomarker of SB. As such, this study provides a foundation for future studies examining the neural substrates of the ACS, allowing for integration of previous findings of higher male vulnerability for death by suicide and higher female vulnerability for non-fatal SB. Importantly, since the ACS is a multidimensional construct, identification of neural regions involved in each of the individual constructs provides the basis for their underlying biosignatures.

### ACS Network

Several brain regions demonstrated consistent activation during imaging studies examining psychological constructs thought to be related to the IPTS dimensions. While no previous meta-analyses exist that combine all these constructs of the ACS, some studies have reported ALE analyses of individual constructs. For example, a meta-analysis of gender differences in emotional processing has been reported earlier (55) and is consistent with what we found. There were no regions commonly activated by all four conditions. Fearlessness about death and pain tolerance were not correlated with one another in Witte et al. (30) and, thus, could be said to develop somewhat independently. Thus, it stands to reason that distinct neural areas might be associated with some of the constructs that contribute to acquired capability.

Our data demonstrate that males and females exhibited similar sub-lobar neural network consisting of bilateral putamen, bilateral claustrum, bilateral insula, right caudate, and right thalamus identified under two or more of the IPTS dimensions. This network of brain regions represents a sub-lobar nucleus of regions at the crossroads of emotion and cognitive processing, with functional contributions to both systems. Even so, there were notable differences in the functional network identified for males and females. The primary motor cortex and premotor cortex pertaining to bilateral precentral gyrus and right mid frontal gyrus (also referred to as dorsolateral prefrontal cortex) along with regions in cerebellum were activated exclusively in males, whereas females exhibited activations restricted to limbic system regions, such as the amygdala and cingulate cortex, commonly known to be involved in emotion formation and processing (56). The utilization of motor areas in the ACS network in males may imply that, if the ACS network is activated in males, there are greater chances of them executing the action as intended, in contrast to females. The inclusion of emotionally reactive regions in the ACS network in females supports suicidal ideation but lack of motor regions engaged in the network might be the reason of females having lower chances of implementing a lethal attempt. Additionally, while males demonstrated bilateral caudate activation, females demonstrated right caudate utilization.

### Depression Network

Several meta-analysis studies have been previously conducted to investigate depression (57–59), and our results are in general agreement with them. However, to the best of our knowledge, ALE meta-analyses on gender differences in depression have not been reported before, and hence, this is a contribution of the present work. In our meta-analysis study, the depression network identified for both males and females consisted of bilateral putamen, left claustrum, bilateral insula, right caudate, and left cingulate gyrus. In spite of the commonly activated regions, depression also exclusively activated some regions specific to either males or females. For males, the caudate that has been known for its involvement in reward processing was identified not only in the right, but also the left hemisphere. For females, we identified the right inferior frontal gyrus, which has been notably linked to depression in several research studies and serves as a target for transcranial magnetic stimulation (60). Additionally, females demonstrated activations in numerous regions, such as anterior and posterior cingulate cortex, which have been implicated to be the neural substrates underlying the vulnerability to SB or suicidal ideation (61). This might explain females being at a higher risk of depression, suicidal ideation, and non-lethal SB, than males.

### Neurofunctional Network Supporting Both ACS and Depression

Regions common to ACS and depression network were designated as ACS–depression seeds, separately in males and females. Using meta-analytic connectivity modeling, we obtained regions coactivated by these seeds in males and females, which we designate as the ACS–depression functional network. Although this network contained some regions that were common to both males and females, there were noteworthy differences in the regions activated in males and females. Such regions (Table 5) were found to contain voxels common to both males and females but also had voxels that were exclusive to the male or female networks. This indicates some level of functional parcellation/differentiation in these brain regions. The notable amount of premotor, primary motor, and cerebellar regions engaged in the ACS–depression network in males might point toward the existence of a neural substrate supporting motor action. This could possibly result in higher probability of a fatal outcome in men on account of experiencing suicidal desire, compared to women. Males also engaged a larger ACS–depression functional network than women, which might be a factor responsible for higher lethal SB in men.

### Structural Network for ACS–Depression

The putamen was the only brain region commonly present in the ACS–depression network seeds for males and females. Therefore, this may be a region of particular importance in understanding SB across men and women. This result is consistent with a recent meta-analytic finding that individuals with a history of SB had decreased volumes in the putamen compared to individuals with a history of psychiatric disorders (61). Upon investigation of the fiber trajectories from the putamen seeds in males and females, differential structural connectivity patterns were observed between males and females. The trajectories from the left putamen seed in males projected up to the premotor cortex, medial frontal gyrus, left insula, and thalamus. Premotor cortex (involved in motor planning), medial frontal gyrus [known to play a role in executive mechanisms (62)], and left insula have been previously shown to form a part of the hate circuit (54) that is engaged while experiencing hate toward an individual [notably, insula is also implicated in perception of the degree of real (63) as well as imaginary pain (64); this provides a possible link as to why it may be involved in the hate circuit], which may be relevant to self-hatred. Engaging a large portion of hate circuit and, more importantly, direct structural connections among the regions in the hate circuit might strengthen the intent of suicide and its execution in males, especially given the involvement of the premotor cortex. While the projections from the left putamen in females were localized, males had projections from the right putamen seed extending up to the anterior cingulate and thalamus. The anterior cingulate has been implicated in a previous study (61) to be a neural substrate underlying suicidal ideation. This might be a reason for higher vulnerability of women for suicidal ideation and non-fatal attempts. The regions identified in the functional ACS–depression network and the structural connectivity of the ACS–depression seeds may be the key to understanding gender differences in the rates of fatal and non-fatal SB.

It is instructive to examine existing literature on postmortem studies from completed suicide with our findings. Furczyk et al. (65) have provided a comprehensive review of postmortem studies in completed suicide. They show that genetics, neurotransmitters, cell signaling, and markers of neural plasticity are altered in individuals who have committed suicide. They report alterations in the above metrics in some of the same regions that we have found. Specifically, (i) Table 1 in Furczyk et al. (65) lists all studies which have shown altered genetic expression and transcription in specific brain regions, notably the prefrontal cortex in postmortem studies, (ii) Table 2 in Furczyk et al. (65) lists all postmortem studies, which have shown alterations in various neurotransmitter systems, including serotonergic, noradrenergic, dopaminergic, glutamatergic, GAMA-ergic, and endocannabinoid systems. These systems comprises regions implicated in our study, notably the frontal cortex, putamen, amygdala, hippocampus, and other areas of the limbic system, (iii) Table 3 in Furczyk et al. (65) lists all postmortem studies, which have shown alterations in various cell signaling systems, including adenylate cyclase, phospholipase C, and cytokines with location specificity mainly in the prefrontal cortex and hippocampus, (iv) Table 4 in Furczyk et al. (65) lists all postmortem studies, which have shown changes in neural plasticity in suicide victims; changes in neurotrophic factors seems to be localized to the prefrontal cortex and hippocampus, while changes in polyamines seem to be global. While some of the studies cited by Furczyk et al. were carried out in only males or females, we could not find any reports where in sex differences have been investigated in postmortem studies.

The results of our study have important implications for the construct validity of ACS. The vast majority of research on ACS has utilized either self-report measures or assessments of physical pain tolerance; to our knowledge, this is the first investigation of neural substrates that may underlie ACS. By constructing a layout of the neural networks, research will also be enhanced as efforts are directed toward answering more complex questions about how neuronal networks contribute to SB.

Some limitations of the current study are noteworthy. First, and perhaps most importantly, we did not investigate SB as an outcome variable. Thus, future research is needed to demonstrate a link between the proposed ACS network and fatal suicide attempts as an outcome. Related to this point, we would like to emphasize that the proposed ACS network is not put forward as a neural substrate for suicide *per se*; rather, we are proposing that it underlies endophenotypes for suicide (i.e., emotional stoicism, sensation-seeking, pain tolerance, and fearlessness about death), which themselves may be endophenotypes for suicide. Given the complexity involved in SB, we are not proposing pure biological determinism, as we recognize the strong influence of environmental risk factors on SB. Second, there are few imaging studies investigating the specific constructs of ACS (i.e., emotional stoicism, sensation-seeking, pain tolerance, and fearlessness of death). Therefore, we performed meta-analyses on emotion, reward, pain, and fear, respectively (since they represent a super-set of the original constructs of ACS), with the assumption that the voxels activated in more than two of the four conditions would be related to ACS. This illustrates one of the weaknesses of meta-analyses. Nevertheless, it is useful to make this assumption as an attempt to generate a hypothesis about the underlying neural substrates of ACS so that future experimental studies may perform experiments to confirm or deny the hypotheses generated by this initial attempt. Similarly, our definition of the ACS network is not ideal (i.e., >2 of the IPTS dimensions), and as the databases become larger, we may be able to refine this definition. Third, we did not investigate all components of the IPTS. Thus, although our results demonstrate that the ACS network is distinct from the depression network, future research is needed to investigate whether distinct neural substrates underlie ACS versus thwarted belongingness and perceived burdensomeness. Fourth, we did not investigate whether the ACS network we obtained generalizes across different forms of psychopathology (e.g., bipolar disorder versus major depressive disorder). This is an important avenue for future research. Fifth, deactivations were not considered in these meta-analyses for the following reasons: (a) deactivations are not as frequently reported as activations, and (b) the neural basis of fMRI-based deactivation is yet unclear, i.e., there is still a debate whether deactivations in fMRI are indeed caused by GABAergic inhibition (66, 67). Finally, the limitations of ALE-based meta-analysis, which have been discussed before, also apply to this study (43, 48, 49, 68). For example, we considered studies that examined the psychological construct “fear” and have considered it as equivalent to fearlessness of IPTS dimension. While fear is a basic emotion and involves predominantly limbic structures, fearlessness is an acquired ability and likely involves inputs from higher cortical structures. A similar case can be made with respect to pain and pain tolerance. Given that a Sleuth search on fearlessness (or pain tolerance) returns no relevant papers, we have to assume that both fear and fearlessness are mediated by the same neural structures. While this assumption may not be entirely true, it may be fine since we are using the meta-analyses as a tool for generating hypotheses to be tested with real data, rather than making mechanistic conclusions solely based on the meta-analyses. Despite these limitations, this study provides a useful foundation for future studies of gender differences in the neural basis of SB.

## Author Contributions

GD, TW, and JR conceived the study. MB performed data analysis under the guidance of GD and JR. GD, MB, TW, and JR wrote the paper.

## Conflict of Interest Statement

The authors declare that the research was conducted in the absence of any commercial or financial relationships that could be construed as a potential conflict of interest. The reviewer CT and handling Editor declared their shared affiliation, and the handling Editor states that the process nevertheless met the standards of a fair and objective review.
